# A Spider in the Spider’s View: Behçet’s Disease-related Giant Coronary Aneurysm

**DOI:** 10.4274/balkanmedj.galenos.2020.2019.12.7

**Published:** 2020-02-28

**Authors:** Servet Altay, Muhammet Gürdoğan, Mustafa Adem Yılmaztepe

**Affiliations:** 1 Department of Cardiology, Trakya University School of Medicine, Edirne, Turkey

A 47-year-old man with a history of Behçet’s disease was admitted to the cardiology clinic with exertional dyspnea of three months’ duration. His physical examination revealed a blood pressure and heart rate of 120/70 mmHg and 81 bpm, respectively, with normal heart and pulmonary auscultation.

Blood biochemical parameters, including troponin I and D-dimer tests, were in the normal ranges. Serial electrocardiography (ECG) findings were normal. The exercise ECG test, which was performed for typical chest pain, showed a 1.5 mm ST-segment depression in leads V4-V6. His coronary angiography revealed a large coronary aneurysm at the left anterior descending (LAD) and diagonal artery (DA) border. On the left anterior oblique caudal view (or spider view), his left coronary system resembled a spider (Figure 1). Coronary artery by-pass surgery was performed on two vessels with the LAD-left internal mammary artery and DA-saphenous graft for treating the severe aneurysm and vascular findings. There were no adverse events at the long-term follow-up of the patient. Written informed consent was obtained from the patient for publication.

Behçet’s disease is an autoinflammatory disease that is characterized by recurrent oral and urogenital ulcers (1). Genetic and environmental factors may participate in the pathogenesis of Behçet’s disease (2). Although arterial manifestations are uncommon, patients may present with femoral and pulmonary arterial aneurysm, aortitis, and arterial thrombosis. Coronary artery aneurysm remains a very rare pathology in Behçet’s disease, reported in approximately 0.5% of patients (3,4). Its etiology involves inflammatory endarteritis of the vasa vasorum. This inflammation leads to the destruction of the tunica media and fibrosis, with resulting arterial wall weakening and aneurysm formation (4). Patients with Behçet’s disease should be followed up in the cardiology clinic, and the management of cardiac risk factors is necessary. Physicians should be aware of the complications of this disease.

## Figures and Tables

**Figure 1:**
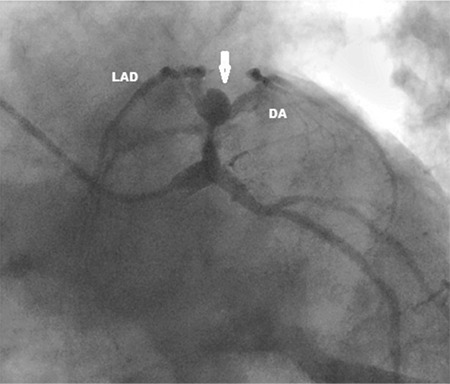
Coronary angiography reveals a large coronary aneurysm at the left anterior descending and diagonal artery border (arrow). On the left, the anterior oblique caudal view (or spider view), the left coronary system resembles a spider. DA: diagonal artery, LAD: left anterior descending
